# Machine Learning-Based Prediction of In-Hospital Mortality in Severe COVID-19 Patients Using Hematological Markers

**DOI:** 10.1155/cjid/6606842

**Published:** 2025-05-12

**Authors:** Rongrong Dong, Han Yao, Taoran Chen, Wenjing Yang, Qi Zhou, Jiancheng Xu

**Affiliations:** ^1^Department of Laboratory Medicine, First Hospital of Jilin University, Changchun 130021, Jilin, China; ^2^Department of Pediatrics, First Hospital of Jilin University, Changchun 130021, Jilin, China

**Keywords:** COVID-19, death, hematological parameters, machine learning, prediction model

## Abstract

**Background:** The mortality rate is very high in patients with severe COVID-19. Nearly 32% of COVID-19 patients are critically ill, with mortality rates ranging from 8.1% to 33%. Early risk factor detection makes it easier to get the right care and estimate the prognosis. This study aimed to develop and validate a model to predict the risk of mortality based on hematological parameters at hospital admission in patients with severe COVID-19.

**Methods:** The study retrospectively collected clinical data and laboratory test results from 396 and 112 patients with severe COVID-19 in two tertiary care hospitals as Cohort 1 and Cohort 2, respectively. Cohort 1 was to train the model. The LASSO method was used to screen features. The models built by nine machine learning algorithms were compared to screen the best algorithm and model. The model was visualized using nomogram, followed by trend analyses, and finally subgroup analyses. Cohort 2 was for external validation.

**Results:** In Cohort 1, the model developed by the LR algorithm performed the best, with an AUC of 0.852 (95% CI: 0.750–0.953). Five features were included in the model, namely, D-dimer, platelets, neutrophil count, lymphocyte count, and activated partial thromboplastin time. The mode had higher diagnostic accuracy in patients with severe COVID-19 > 65 years of age (AUC = 0.814), slightly lower than in patients with severe COVID-19 ≤ 65 years of age (AUC = 0.875). The ability of the model to predict the occurrence of mortality was validated in Cohort 2 (AUC = 0.841).

**Conclusions:** The risk prediction model for mortality for patients with severe COVID-19 was constructed by the LR algorithm using only hematological parameters in this study. The model contributes to the timely and accurate stratification and management of patients with severe COVID-19.

## 1. Introduction

Coronavirus disease 2019 (COVID-19) is an infectious disease caused by severe acute respiratory syndrome coronavirus 2 (SARS-CoV-2). Nearly 32% of COVID-19 patients are critically ill, with mortality rates ranging from 8.1% to 33% [1]. After 1 week from the time of onset, severe COVID-19 patients frequently experience respiratory distress and hypoxemia. In extreme situations, patients with severe COVID-19 progress rapidly to acute respiratory failure, acute respiratory distress syndrome, metabolic acidosis, coagulopathy, septic shock, and even death [2]. Early risk factor detection makes it easier to get the right care and estimate the prognosis. Therefore, it is necessary to make a judgment about the patient's condition based on laboratory findings.

The clinical traits of COVID-19 patients have demonstrated that a variety of laboratory factors are associated with the severity of the illness. Although a few studies have reported the serum levels of ferritin, D-dimer, lactate dehydrogenase, C-reactive protein, troponin I, procalcitonin, and IL-6 of patients with COVID-19, which have all been proposed to carry prognostic significance [3]. However, these studies have largely been cross-sectional and cannot accurately depict the entire course of the disease. In addition, several earlier research studies lacked external validation and relied on complicated tests for prediction [4]. The methods to anticipate the course of a patient's condition quickly and affordably are currently lacking. Blood intimately interacts with various human tissues and cells, and it can reveal a variety of information [5]. A complete blood count is the most common test done in clinical practice on hospitalized patients, which is easy, quick, and reasonably priced [6, 7]. Blood count parameters have been studied to correlate with the severity and mortality of COVID-19 cases [8]. Meanwhile, a study report that severe COVID-19 is commonly complicated with coagulopathy [9], and elevated levels of D-dimer and fibrin/fibrinogen degradation products (FDPs) are associated with a poor prognosis in severe COVID-19 [10, 11].

Machine learning (ML) is a subfield of artificial intelligence that encapsulates statistical and mathematical algorithms that enable fact-finding and complex decision-making [12]. ML algorithms have been explored in myriad fields of COVID-19, such as detection of outbreaks, rapid diagnosis, severity risk prediction, and prognosis projection [13–17]. Recent studies have established predictive models employing laboratory test data based on several COVID-19 patient features and outcomes, such as the requirement for intensive care, mechanical ventilation, and death [18–20].

The data on the hematological parameters of severe COVID-19 patients treated at the First Hospital of Jilin University between December 1, 2022, and January 31, 2023, will be used in this study to conduct a retrospective study and utilize ML techniques to analyze the mortality factors of COVID-19 patients. In addition, the same predictor factors, outcome-defining techniques, and measures will be used in the project to collect clinical data from another hospital concurrently for external validation. A straightforward and trustworthy mortality risk prediction model for patients with severe COVID-19 will be investigated in this study. This model will assist doctors in managing COVID-19 patients, predicting disease trajectories, allocating healthcare resources effectively, and enhancing patient prognosis.

## 2. Methods

### 2.1. Study Subjects

Cohort 1 (model of building and internal validation): severe COVID-19 patients who received intensive treatment at the First Hospital of Jilin University between December 1, 2022, and January 31, 2023, and had a known outcome (discharge or death), were included to establish a mortality risk prediction.

Cohort 2 (model of external validation): severe COVID-19 patients who received intensive treatment at Meihekou City Central Hospital between December 1, 2022, and January 31, 2023, and had a known outcome (discharge or death), were included for external validation.

Inclusion criteria for patients with COVID-19: (1) age ≥ 18 years; (2) a confirmed case of COVID-19 was defined as a real-time reverse transcription polymerase chain reaction (RT-PCR) test of a nasopharyngeal swab; and (3) blood tests were carried out before or within 24 h of the initial positive RT-PCR finding.

Exclusion criteria for patients with COVID-19: (1) mild and common patients; (2) pregnant patients; (3) patients who are readmitted or released for unique circumstances, such as dialysis; and (4) people with documented immunodeficiencies, such as those using immunosuppressive drugs, those with active malignancies, and those who have autoimmune diseases.

### 2.2. Diagnostic Criteria

Clinical typing according to the Clinical Treatment Program for Novel Coronavirus Pneumonia (Trial 9 Edition) issued by the General Office of the National Health Commission [21]: (1) mild cases: mild clinical symptoms, no pneumonia manifestations in imaging; (2) common cases: fever, respiratory tract, and other symptoms, pneumonia manifestations can be seen in imaging; and (3) severe type: meet any of the following: respiratory distress, RR ≥ 30  times/min; in resting state, it means oxygen saturation  ≤  93%; arterial oxygen partial pressure/fraction of inspired oxygen  ≤  300 mmHg; patients whose lung imaging showed significant progression of the lesions within 24–48 h and whose lung lesions accounted for > 50% of the lung area were treated according to the management protocol for severe cases.

### 2.3. Data Collection

The basic information and medical history information of the study subjects were collected from the medical history system; the basic information, clinical diagnosis, and first test data of the study subjects were collected from the laboratory information system. The ID number was used as the unique identification of the study subject. The following clinical data were collected for all patients: (1) general information: gender, age, and duration of hospitalization; (2) medical history: history of endocrine system disease, cerebrovascular disease, cardiovascular disease, and others; (3) first symptoms: cough, fever, fatigue, myalgia, pleuritic chest pain, etc.; (4) outcome: survived or nonsurvived; and (5) results of laboratory tests on admission. Data exclusion and padding: exclude tests with missing rates > 30%. Based on the data distribution characteristics, we select the filling method (median, mean, and plural) that can represent the central tendency of the variables.

### 2.4. Research Design

**Figure 1:**
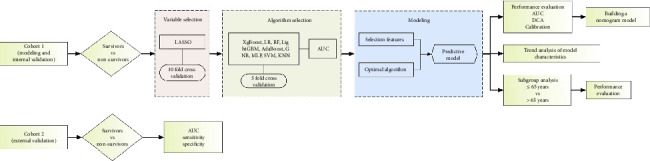
Flowchart of machine learning to build the predictive model. XgBoost, extreme gradient boosting; LR, logistic regression; RF, random forest; LightGBM, lightweight gradient boosting machine learning; AdaBoost, adaptive boosting algorithm; GNB, gaussian plain bayes; MLP, neural networks; SVM, support vector machines; KNN, k-nearest neighbor; AUC, area under the curve; ROC, receiver operator characteristic curve; DCA, decision curve analysis.

Least Absolute Shrinkage and Selector Operation (LASSO) was used to screen Cohort 1 for characteristics. The nine ML algorithms, namely, extreme gradient boosting (XgBoost), logistic regression (LR), random forest (RF), lightweight gradient boosting machine learning (LightGBM), adaptive boosting algorithm (AdaBoost), gaussian plain bayes (GNB), neural networks (MLP), support vector machines (SVM), and K-nearest neighbor (KNN), were used to build the prediction models and compare the results of internal 5-fold cross-validation of the nine algorithms. The ML algorithm with the best performance was selected as the algorithm for subsequent model building and validation, and the selected features from the optimal algorithm were used as the features for subsequent model building. External validation of the model was performed in Cohort 2 (Figure 1).

### 2.5. Statistical Analysis

Excel 2016 was used to store and manage the data, and SPSS 22.0 was used for statistical analysis. Feature selection and model construction were carried out using the Deepwise & Beckman Coulter DxAI platform (https://dxonline.deepwisecom). The receiver operating characteristic (ROC) curve was used to assess the classification effectiveness of the model; the calibration curve was used to assess the agreement between the model prediction probabilities and the sample probabilities; and decision curve analysis (DCA) was used to assess the clinical benefit of the model. GraphPad Prism 9.5 was used for trend analysis. Normally distributed variables were expressed as the mean ± standard deviation, and non-normally distributed variables were expressed as median (Q25 and Q75), and the Student's *t*-test, Mann–Whitney *U* test, ANOVA, or Kruskal–Wallis H test were used to compare the distribution of variables between groups. Categorical variables were expressed as composition ratios, and the Pearson χ^2^ test or Fisher exact test was used to compare the distribution between groups, with a two-sided *p* < 0.05 as a statistically significant difference.

## 3. Results

### 3.1. Baseline Characteristics

Cohort 1 enrolled a total of 396 patients with severe COVID-19, including 290 survivors and 106 nonsurvivors. Cohort 2 enrolled a total of 112 severe COVID-19 patients, including 83 survivors and 29 nonsurvivors. In Cohort 1, the median age was 71 years and the median number of days of hospitalization was 6 days, with females comprising 42.68% of the cohort. In Cohort 2, the median age was 72 years and the median number of days of hospitalization was 13 days, with females comprising 34.82% of the cohort. The most common comorbidity in both Cohort 1 and Cohort 2 was endocrine system disease, and the most common clinical symptom was fever in both. Endocrine system disease, cerebrovascular disease, fever, and pleuritic chest discomfort were among the clinical characteristics that were significantly different between survivors and nonsurvivors in Cohorts 1 and 2 (*p* < 0.05) (Table 1).

### 3.2. Selection of Model Features and Optimal Algorithms

**Figure 2:**
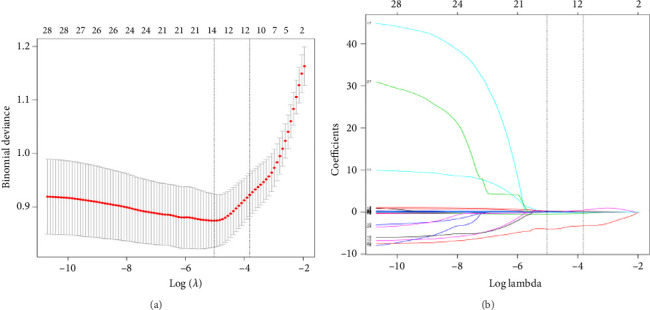
Clinical feature selection using LASSO regression. (a) Tuning parameter (*λ*) selection in the LASSO model used 10 fold cross validation via minimum criteria. Dotted vertical lines were drawn at the optimal values by using the minimum criteria and the 1 standard error of the minimum criteria (the 1-SE criteria). (b) LASSO coefficient profiles of the 29 clinical features. A coefficient profile plot was produced against the log (*λ*) sequence.

Fifty features were ultimately chosen after screening the test items and deleting features with a missing rate greater than 30%: 29 hematologic features and 21 other features (10 liver function tests, 4 ions, 3 kidney function tests, and 4 heart function tests). Twenty-nine hematological features were subjected to feature selection by the LASSO method, and 11 features to be selected for modeling were finally included: hemoglobin (HGB), mean platelet volume (MPV), D-dimer (DD), fibrinogen (FBG), platelets (PLT), neutrophil count (N1), lymphocyte count (L1), percentage of neutrophils (N), lymphocyte percentage (L), fibrinogen degradation product (FDP), and activated partial thromboplastin time (APTT) (Figure 2).

The internal 5 fold cross validation results of the nine algorithms found that LR showed the highest AUC of 0.835 (95% CI: 0.93–0.96) for predicting. Therefore, the algorithm used for subsequent modeling was LR, and the features used for subsequent modeling were identified as the five features with the highest feature importance in the LR algorithm, namely, DD, N1, L1, PLT, and APTT.

### 3.3. Building a Predictive Model and a Nomogram Model

**Figure 3:**
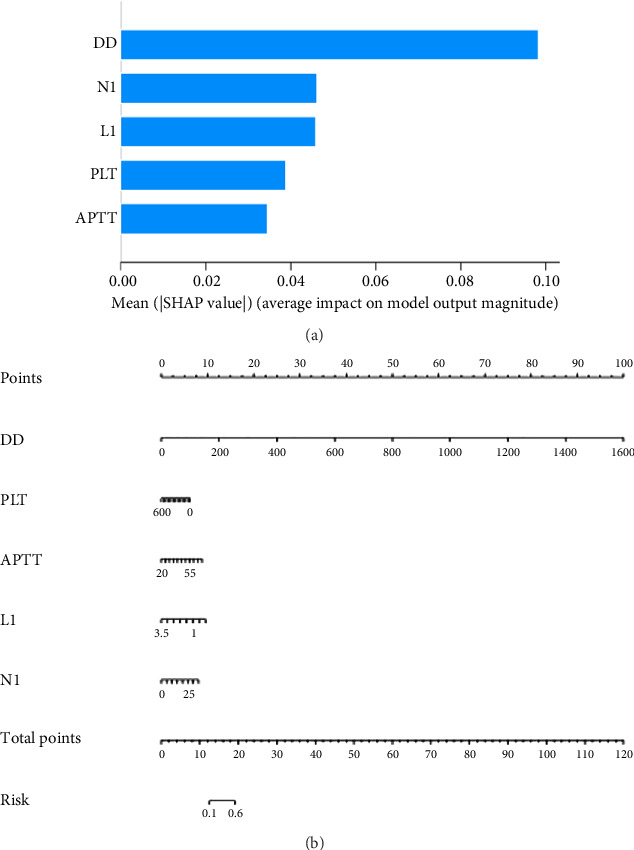
(a) The varying importance of the functions included in the mortality risk early warning model. (b) Nomogram to predict the mortality risk of severe COVID-19.

**Figure 4:**
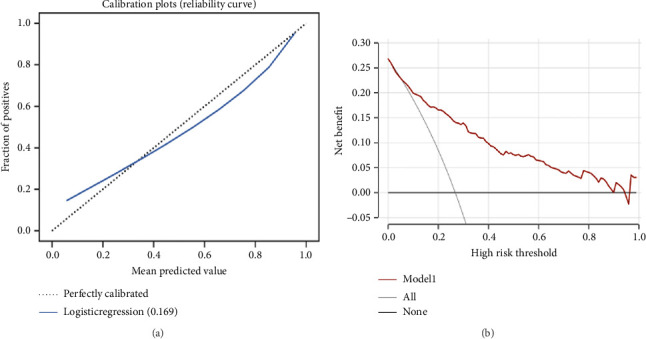
In the internal validation set, the model built by the LR algorithm was evaluated for its ability to predict the risk of death occurring in patients with severe COVID-19. (a) Calibration curve analysis. (b) Decision curve analysis.

The LR method was used to build a mortality risk prediction model based on five model features, and a nomogram was produced to represent the model (Figure 3). The model's most crucial component was DD, which was then followed by N1, L1, PLT, and APTT in that order. The model's capacity for prediction was evaluated in the study. Internally validated ROC curve results showed that the model had excellent classification ability in predicting the risk of death occurring in severe COVID-19 patients (AUC =0.852 [95% CI: 0.750–0.953]), sensitivity = 0.820, specificity = 0.768); the calibration curve showed that the model's sample probabilities were in good agreement with the predicted probabilities; and the results of DCA showed that the model had a high clinical benefit (Figure 4).

### 3.4. Analyses of Trends in Model Characteristics and Age Subgroups

**Figure 5:**
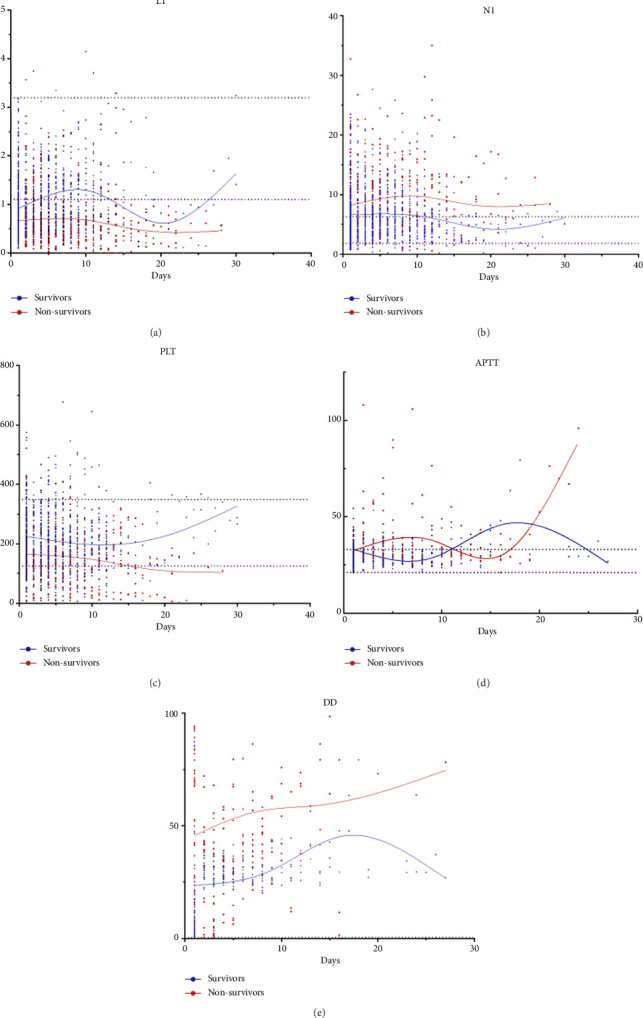
Trends in the model's parameters. (a) Lymphocyte count. (b) Neutrophil count. (c) Platelets. (d) Activated partial thromboplastin time. (e) D-dimer.

Throughout the course of illness, the average values of PLT and L1 were lower, and the average values of N1 and D-dimer were higher among nonsurvivors compared with those survivors. The average values of APTT were observed to be substantially higher among nonsurvivors compared with survivors near the end of the research period. On Day 15, it was observed that the average values of PLT for nonsurvivors gradually dropped to below the RI's lower limit, whereas the average values of PLT for survivors steadily increased within the RI's range. The average values of L1 in nonsurvivors were found persistently below the lower limit of the RI, while the average values of survivors were found close to the lower limit of the RI for the first 20 days before increasing after hitting a low point on Day 20. On Day 10, it was observed that the average values of L1 in survivors started to fall and fell within the RI range, with the average values of L1 in nonsurvivors remaining continuously beyond the RI's upper limit. The average values of DD in both survivors and nonsurvivors were always observed over the upper RI line, with the average values of DD in survivors exhibiting a *U*-shaped pattern that peaked on Day 17. For the first 11 days, the average values of APTT were higher among nonsurvivors compared to those survivors and above the upper RI limit. However, after Day 11, it was found that the average values of APTT decreased to within the RI range, increased quickly after Day 15, and were higher than the nonsurvivors after Day 20. The opposite was observed among nonsurvivors, peaking on Day 17 and then progressively falling (Figure 5).

In Cohort 1, the diagnostic effectiveness of risk prediction models for mortality was compared across age groups. The model showed very excellent predictive ability for patients with severe COVID-19 under 65 years old (AUC = 0.875, sensitivity = 1.000, and specificity = 0.854) and high predictive ability for patients with severe COVID-19 over 65 years old (AUC = 0.814, sensitivity = 0.730, and specificity = 0.683).

### 3.5. External Verification

In Cohort 2, 112 patients with severe COVID-19 were enrolled, including 83 survivors and 29 nonsurvivors. The ROC curve results showed that the model has stable and excellent death risk prediction ability (AUC = 0.841, sensitivity = 0.790, and specificity = 0.821).

## 4. Discussion

This study had the following innovative findings: (1) a variety of ML algorithms were used for comparison to select the optimal algorithm, which provided a stable foundation for modeling and a reliable method for subsequent model selection. (2) The discrimination, calibration, and clinical usefulness of the model were evaluated using training and external validation cohorts, which could more comprehensively demonstrate the prediction ability of the model based on hematological parameters. (3) Routine hematological indicators alone could be used to realistically predict the probability of death at the time of patient admission.

### 4.1. Modeling and Trend Analysis

In this study, the optimal algorithm LR was selected from among nine ML algorithms. Compared with the other algorithms, LR avoided overfitting in unbalanced datasets and could be tuned for unbalanced datasets. A ML model consisting of five biomarkers, namely, DD, PLT, N1, L1, and APTT, was proposed in this study. Abnormal coagulation is a significant factor in the COVID-19 patient's prognosis. Analyses of the laboratory results of patients with COVID-19 by some researchers reveal that severe patients are frequently found to experience extended PT, elevated D-dimer levels, and low fibrinogen levels [22]. One of the breakdown products of fibrinolysis, DD, was found to be significantly elevated in the current study throughout the course of the disease, indicating that individuals might be experiencing excessive fibrinolysis [23]. In addition, most of the patients were admitted with APTT levels within the normal range, which is associated with the hypercoagulable state that is common in early COVID-19. It has been discovered that coagulation factor deficiency may be the cause of the prolonged lengthening of APTT in nonsurvivors during the latter stages of the disease [24].

Elevated NLR, along with reduced platelet levels, are more likely to exist in critical patients compared with those in mild and common patients [8]. An increased NLR is an increase in neutrophil count and a decrease in lymphocyte count. In the innate immune system, neutrophils play a crucial role, while lymphocytes are crucial for the inflammatory response. Therefore, the imbalance of the innate immune system may indicate a high NLR and is a potential marker in many infections [25]. According to studies, a decrease in platelet count indicates unfavorable prognostic characteristics in patients with COVID-19 [26, 27]. SARS-CoV-2 infection may decrease platelet production by the bone marrow; platelet aggregation in the lungs may increase platelet consumption; and destruction of platelets by the immune system may increase [28]. As described by Bai et al. [14], nonsurvivors were found to have decreased platelet counts during hospitalization and developed significant DIC [29].

### 4.2. Model of Validation

The study relied on the retrospective collection of clinical data. However, ML techniques were used in the study, which increased the generalizability of the results by obtaining data from a large cohort of patients admitted to tertiary care. The proposed model for ML was reliably externally validated using an external database, as well as showing good performance. Age has been identified as strongly associated with the incidence of death in COVID-19 patients, according to a number of observational studies [30, 31]. The model established in this study was verified to have high accuracy for the risk evaluation of mortality in both elderly and nonelderly groups. In conclusion, the model can be used by clinicians to predict the risk of mortality in patients with severe COVID-19 and take effective treatment in advance.

This study had several limitations: (1) the AUC values were lower than 0.9, and more cases should be recruited to optimize this prediction model for more precise forecasting. (2) Patients affected by liver, cardiovascular, renal, or malignant diseases or other previous comorbidities and/or conditions that could bias the study were not excluded from this study. (3) The patients included were primarily local residents from Jilin Province, China. The predictive performance of the models had not been investigated in other regions or ethnicities.

## 5. Conclusion

This study analyzed 508 patients with severe COVID-19 and developed a mortality risk prediction model based on hematological parameters using ML algorithms. Through comparison, LR was selected as the optimal algorithm, and five key features were identified: DD, PLT, N1, L1, and APTT. The model exhibited high predictive accuracy for both patients aged ≤ 65 and those > 65 years. The construction of this model provides a timely and accurate stratification tool for the management of patients with severe COVID-19, which may assist in improving clinical decision-making and patient outcomes. Furthermore, the findings of this study underscore the necessity for further validation of the predictive performance of ML models across different regions and populations to ensure their broad applicability and effectiveness.

## Figures and Tables

**Table 1:** Clinical baseline characteristics.

Characteristics	Queue 1				Queue 2			
	Total (*n* = 396)	Survivors (*n* = 290)	Nonsurvivors (*n* = 106)	*p*	Total (*n* = 112)	Survivors (*n* = 83)	Nonsurvivors (*n* = 29)	*p*
Sex, *n* (%)								
Female	169 (42.67)	132 (45.52)	37 (34.91)	0.059	39 (34.82)	32 (38.55)	7 (24.14)	0.161
Age, year, median (IQR)	71.00 (62.00, 82.00)	69.00 (60.00, 78.00)	80.00 (69.00, 87.00)	< 0.001	71.91 ± 11.62	70.84 ± 11.79	74.96 ± 10.55	0.102
Hospitalized time, day, median (IQR)	6 (3, 10)	7 (4, 11)	5 (2, 8)	0.00	13 (10, 17)	14 (10, 17)	10 (8, 15)	0.01
Any comorbidities *n* (%)								
Endocrine system disease	212 (53.54)	139 (47.93)	73 (68.87)	< 0.001	57 (50.90)	34 (40.96)	23 (79.31)	< 0.001
Cardiovascular disease	203 (51.26)	141 (48.62)	62 (58.50)	0.082	54 (48.21)	28 (33.74)	26 (89.66)	< 0.001
Cerebrovascular disease	146 (36.87)	96 (33.10)	50 (47.17)	0.01	41 (36.61)	21 (25.30)	20 (68.97)	< 0.001
Others	153 (38.64)	107 (36.90)	46 (43.40)	0.24	49 (43.75)	38 (45.78)	11 (37.93)	0.463
Chief complaint *n* (%)								
Cough	181 (45.71)	130 (44.83)	51 (48.11)	0.561	49 (43.75)	37 (44.58)	12 (41.38)	0.765
Fever	273 (68.94)	182 (62.76)	91 (85.85)	< 0.001	85 (75.90)	59 (71.08)	26 (89.66)	0.044
Fatigue	115 (29.04)	76 (26.21)	39 (36.79)	0.04	27 (24.11)	17 (20.49)	10 (34.48)	0.129
Myalgia	99 (25.00)	67 (23.10)	32 (30.19)	0.149	45 (40.18)	21 (25.30)	24 (82.76)	< 0.001
Pleuritic chest pain	63 (15.91)	37 (12.76)	26 (24.53)	0.005	33 (29.46)	11 (13.25)	22 (75.86)	< 0.001
Others	169 (42.68)	119 (41.03)	50 (47.17)	0.274	51 (45.54)	38 (45.78)	13 (44.83)	0.929
Coagulation parameters, median (IQR)								
PT: 9–13 s	12.50 (11.80, 13.40)	12.30 (11.70, 12.90)	13.00 (12.40, 13.80)	< 0.001	13.00 (12.40, 14.10)	13.00 (12.40, 14.10)	13.00 (12.30, 14.10)	0.881
INR: 0.8–1.2	1.06 (0.99, 1.14)	1.04 (0.99, 1.10)	1.11 (1.04, 1.17)	< 0.001	1.15 (1.10, 1.25)	1.14 (1.10, 1.25)	1.19 (1.10, 1.24)	0.625
APTT: 21–33 s	27.50 (25.70, 30.21)	26.90 (25.40, 29.60)	29.30 (26.80, 31.30)	< 0.001	31.30 (27.60, 34.80)	30.50 (27.30, 34.20)	33.50 (28.70, 38.50)	0.009
TT: 11–21s	17.10 (16.40, 18.00)	17.00 (16.40, 18.00)	17.50 (16.50, 18.20)	0.03	16.88 ± 1.57	16.90 ± 1.41	16.81 ± 1.95	0.791
FBG: 1.8–4 g/L	4.16 (3.03, 5.03)	4.02 (2.99, 4.99)	4.48 (3.08, 5.30)	0.12	4.84 (3.44, 6.22)	4.54 (3.44, 5.87)	5.98 (4.68, 7.38)	0.002
DD: 0–0.5 mg/L	1.09 (0.51, 4.24)	0.79 (0.42, 1.85)	6.74 (1.59, 67.90)	< 0.001	1.46 (1.06, 2.63)	1.24 (0.89, 1.88)	2.93 (1.87, 6.53)	< 0.001
FDP: 0–5 μg/mL	3.21 (2.50, 12.03)	2.50 (2.50, 5.60)	30.41 (5.91, 30.41)	< 0.001	5.10 (3.80, 7.50)	4.70 (3.70, 7.00)	6.40 (4.30, 8.00)	0.029
Leukocyte parameters, median (IQR)								
WBC: 3.5–9.5 × 10^9^/L	6.92 (5.22, 9.64)	6.40 (4.88, 9.03)	8.32 (6.35, 12.17)	< 0.001	6.63 (4.90, 8.74)	6.46 (5.12, 8.57)	6.69 (4.77, 11.35)	0.537
L1: 1.1–3.2 × 10^9^/L	0.84 (0.52, 1.32)	0.95 (0.62, 1.38)	0.59 (0.41, 0.86)	< 0.001	0.77 (0.53, 1.23)	0.90 (0.62, 1.56)	0.61 (0.40, 0.76)	< 0.001
M1: 0.1–0.6 × 10^9^/L	0.49 (0.31, 0.66)	0.48 (0.31, 0.63)	0.48 (0.30, 0.70)	0.53	0.45 (0.27, 0.56)	0.46 (0.31, 0.59)	0.29 (0.15, 0.49)	0.002
N1: 1.8–6.3 × 10^9^/L	5.26 (3.60, 8.26)	4.73 (3.14, 7.45)	7.11 (4.91, 11.05)	< 0.001	5.21 (3.68, 6.40)	4.89 (3.76, 6.24)	5.46 (3.21, 8.92)	0.393
E1: 0.02–0.52 × 10^9^/L	0.01 (0.00, 0.06)	0.02 (0.00, 0.08)	0.00 (0.00, 0.02)	< 0.001	0.09 (0.01, 0.10)	0.09 (0.01, 0.10)	0.09 (0.00, 0.10)	0.516
B1: 0–0.06 × 10^9^/L	0.01 (0.01, 0.02)	0.01 (0.01, 0.02)	0.01 (0.01, 0.03)	0.21	0.04 (0.01, 0.10)	0.03 (0.01, 0.10)	0.06 (0.02, 0.10)	0.599
Erythrocyte parameters, median (IQR)								
RBC: 4.3–5.8/3.8–5.1 × 10^12^/L	4.24 (3.71,4.68)	4.23 (3.71,4.64)	4.27 (3.70,4.86)	0.38	4.23 (3.71,4.65)	4.29 (3.66, 4.69)	4.15 (3.79, 4.62)	0.976
HGB: 130–175/115–150 g/L	130.00 (114.00, 144.00)	129.00 (113.00, 142.00)	132.00 (114.00, 146.00)	0.23	131.00 (114.00, 144.00)	131.00 (114.00, 143.00)	128.00 (116.00, 152.00)	0.651
HCT: 0.4–0.5/0.35–0.45 L/L	0.38 (0.34, 0.43)	0.38 (0.34, 0.42)	0.39 (0.33, 0.43)	0.16	0.39 ± 0.06	0.39 ± 0.06	0.40 ± 0.06	0.272
RDW: 11%–14%	13.00 (12.40, 13.70)	12.80 (12.30, 13.50)	13.20 (12.60, 14.10)	< 0.001	12.60 (12.00, 13.20)	12.50 (11.80, 13.10)	12.80 (12.50, 13.20)	0.114
MCH: 27–34 pg	30.70 (29.50, 31.60)	30.60 (29.50, 31.60)	30.80 (29.80, 31.70)	0.17	31.10 (29.40, 32.30)	30.60 (29.00, 32.30)	31.50 (30.10, 32.30)	0.099
MCV: 82–100 fL	90.70 (87.70, 93.70)	90.50 (87.40, 93.50)	91.20 (88.40, 95.40)	0.12	92.10 (88.20, 94.10)	91.50 (87.70, 93.90)	93.10 (88.60, 96.00)	0.172
MCHC: 316–354 g/L	338.00 (328.00, 344.00)	338.00 (329.00, 343.00)	338.00 (326.00, 346.00)	0.81	335.00 (327.00, 341.00)	336.00 (327.00, 342.00)	334.00 (325.00, 338.00)	0.281
Platelet parameters, median (IQR)								
PLT: 125–350 × 10^9^/L	194.00 (147.00, 262.00)	204.00 (153.00, 278.00)	168.00 (112.00, 214.00)	< 0.001	162.00 (125.00, 218.00)	166.00 (137.00, 236.00)	141.00 (81.00, 173.00)	0.032
MPV: 7–11 fL	10.20 (9.50, 10.90)	10.00 (9.40, 10.70)	10.60 (9.90, 11.20)	< 0.001	9.90 (9.00, 11.00)	9.80 (8.96, 10.60)	10.60 (9.50, 11.30)	0.05
PCT: 0.108%–0.282%	0.20 (0.16, 0.26)	0.21 (0.16, 0.26)	0.18 (0.13, 0.22)	< 0.001	26.04 (22.70, 33.20)	24.70 (21.50, 31.60)	30.30 (25.60, 35.60)	0.015
PDW: 9.7%–17.1%	11.10 (9.80, 12.40)	10.90 (9.60, 12.10)	11.70 (10.40, 13.80)	< 0.001	10.90 (9.70, 12.60)	10.60 (9.40, 12.60)	11.70 (10.60, 13.50)	0.057
Liver function parameters								
DB: 0–6.8 μmol/L, median (IQR)	3.00 (2.10, 5.20)	2.70 (1.90, 4.00)	4.70 (2.90, 7.71)	< 0.001	5.50 (3.60, 7.74)	5.40 (3.40, 7.74)	6.20 (4.40, 7.80)	0.129
IB: 5–20 μmol/L, median (IQR)	9.50 (7.10, 12.50)	8.90 (6.80, 11.90)	10.30 (7.90, 12.71)	< 0.001	4.50 (2.90, 6.50)	5.00 (3.10, 6.90)	3.60 (2.60, 5.37)	0.206
TP: 65–85 g/L, mean (±SD)	63.40 ± 6.80	64.45 ± 6.41	60.50 ± 6.99	< 0.001	60.20 (55.70, 66.00)	61.30 (57.00, 66.70)	58.80 (53.00, 65.20)	0.068
ALB: 40–55 g/L, mean (±SD)	33.72 ± 5.20	34.95 ± 4.97	30.36 ± 4.23	< 0.001	33.51 (31.30, 37.30)	33.90 (31.60, 39.80)	33.51 (28.50, 33.90)	0.01
ALT: 9–50 U/L, median (IQR)	24.90 (15.70, 44.10)	22.80 (15.50, 39.40)	31.20 (19.10, 52.20)	0.00	23.00 (14.00, 33.00)	21.00 (13.00, 33.00)	31.08 (18.00, 33.00)	0.181
AST: 15–40 U/l, median (IQR)	29.40 (20.60, 49.80)	25.80 (19.00, 38.20)	45.70 (29.30, 85.60)	< 0.001	34.00 (21.00, 49.00)	31.00 (20.00, 44.00)	44.00 (26.00, 58.00)	0.051
ALP: 45–125 U/L, median (IQR)	81.60 (64.70, 104.40)	78.60 (63.00, 99.30)	91.80 (69.60, 119.80)	0.01	92.52 (75.00, 92.52)	92.52 (70.00, 92.52)	92.52 (92.52, 92.52)	0.025
TBA: 0–10 umoI/L, median (IQR)	3.90 (2.30, 6.30)	3.60 (2.30, 5.90)	5.30 (2.40, 8.00)	0.02	6.95 (6.95, 6.95)	6.95 (6.95, 6.95)	6.95 (6.95, 6.95)	0.925
GGT: 10–60 U/L, median (IQR)	44.80 (23.30, 87.49)	41.20 (22.90, 80.50)	56.00 (26.70, 91.50)	0.08	76.84 (32.00, 76.84)	72.00 (28.00, 76.84)	76.84 (71.00, 76.84)	0.037
CHE: 4620–11500 U/L, median (IQR)	5568.00 (4548.00, 6969.00)	5973.00 (4888.00, 7254.00)	4809.31 (3835.00, 5621.00)	< 0.001	4951.03 (4364.00, 6199.80)	4951.03 (4462.30, 6633.00)	4951.03 (2929.00, 4951.03)	0.016
Electrolyte parameters, median (IQR)								
K: 3.5–5.3 mmol/L	3.89 (3.58, 4.23)	3.89 (3.58, 4.22)	3.90 (3.56, 4.25)	0.88	3.92 ± 0.50	3.90 ± 0.45	3.99 ± 0.62	0.478
Na: 137–147 mmol/L	137.50 (134.40, 140.20)	137.90 (134.80, 139.90)	136.90 (133.00, 141.20)	0.73	136.00 (132.00, 139.00)	135.000 (131.810, 139.000)	136.00 (133.00, 139.00)	0.351
CL: 98–107 mmol/L	104.30 (101.00, 107.20)	104.10 (101.00, 107.00)	105.30 (100.50, 109.70)	0.19	97.00 (90.00, 101.00)	97.00 (90.00, 100.00)	96.00 (86.00, 104.00)	0.673
Ca: 2.11–2.52 mmol/L	2.06 (1.95, 2.16)	2.09 (1.97, 2.18)	2.00 (1.89, 2.10)	< 0.001	2.03 (1.89, 2.19)	2.05 (1.90, 2.19)	2.00 (1.85, 2.20)	0.611
Kidney function parameters, median (IQR)								
CO2-CP: 22–30 mmol/L	24.50 (21.73, 26.70)	25.20 (22.60, 27.10)	22.47 (20.20, 24.87)	< 0.001	22.80 (20.70, 25.00)	23.60 (21.20, 25.90)	21.00 (17.30, 21.80)	< 0.001
UREA: 3.1–8.8 mmol/L	6.43 (4.71, 9.91)	5.92 (4.45, 8.55)	9.57 (6.17, 16.40)	< 0.001	6.40 (4.40, 11.50)	5.30 (4.10, 9.70)	10.10 (5.60, 17.10)	0.002
CREA: 57–111 mol/L	76.80 (57.80, 108.70)	71.00 (55.40, 91.30)	96.20 (69.60, 162.10)	< 0.001	80.20 (64.10, 129.50)	76.90 (59.70, 103.40)	112.00 (75.80, 189.00)	0.007
Cardiac markers parameters, median (IQR)								
CK-MB: 0–3.38 mg/mL	1.09 (0.50, 2.99)	0.83 (0.41, 1.49)	3.87 (1.62, 5.03)	< 0.001	12.50 (7.20, 18.16)	12.40 (7.60, 18.16)	12.80 (7.20, 17.80)	0.863
cTnt: 0–0.034 μg/L	0.01 (0.01,0.07)	0.01 (0.01,0.03)	0.10 (0.03,1.56)	< 0.001	0.12 (0.01,0.24)	0.12 (0.01,0.24)	0.11 (0.04, 0.27)	0.448
Myo: 0–61.5/121 ng/mL	74.40 (33.90, 223.10)	48.40 (29.30, 110.40)	280.20 (128.70, 676.04)	< 0.001	123.60 (48.10, 232.36)	116.00 (44.60, 232.36)	179.10 (68.50, 240.70)	0.15
NT-proBNP: 0–125pg/mL	1440.00 (238.00, 2200.00)	835.00 (153.00, 2026.26)	3510.00 (875.00, 5135.08)	< 0.001	859.98 (436.00859.98)	859.98 (343.00, 859.98)	859.98 (532.00, 859.98)	0.749

*Note:* cTnT T, cardiac troponin T; P, statistics of differences between survivors and nonsurvivors groups; FBG, fibrinogen; L1, lymphocyte count; M1, monocyte count; N1, neutrophil count; E1, eosinophil count; B1, basophil count; HCT, hematocrit; RDW, red blood cell distribution width; PLT, platelet count; PCT, plateletcrit; ALT, alanine aminotransferase; AST, aspartate aminotransferase; GGT, *γ*-glutamyl transpeptidase; CHE, cholinesterase; K, potassium; Na, sodium; UREA, blood urea nitrogen; NT-proBNP, N-terminal probrain natriuretic peptide; HGB, hemoglobin; CK-MB, serum myocardial enzyme.

Abbreviations: ALB, albumin; ALP, alkaline phosphatase; APTT, activated partial thromboplastin time; B%, basophil percentage; Ca, calcium; Cl, chloride; CO2-CP, carbon dioxide combining power; CREA, creatinine; DB, direct bilirubin; DD, D-dimer; E%, eosinophil percentage; FDP, fibrin degradation product; IB, indirect bilirubin; INR, international normalized ratio; L%, lymphocyte percentage; M%, monocyte percentage; MCH, mean corpuscular hemoglobin; MCV, mean corpuscular volume; MCHC, mean corpuscular hemoglobin concentration; MPV, mean platelet volume; Myo, myoglobin; N%, neutrophil percentage; PDW, platelet distribution width; PT, prothrombin time; RBC, red blood cell; TBA, total bile acid; TP, total protein; TT, thrombin time; WBC, white blood cell.

## Data Availability

The data that support the findings of this study are available on request from the corresponding author. The data are not publicly available due to privacy or ethical restrictions.

## Nomenclature

COVID-19: Coronavirus disease 2019
SARS-CoV-2: Severe acute respiratory syndrome coronavirus 2
ML: Machine learning
LASSO: Least absolute shrinkage and selector operations
XgBoost: Extreme gradient boosting
LR: Logistic regression
RF: Random forest
LightGBM: Lightweight gradient boosting machine learning
AdaBoost: Adaptive boosting algorithm
GNB: Gaussian plain Bayes
MLP: Neural networks
SVM: Support vector machines
KNN: K-nearest neighbor
PT: Prothrombin time
INR: International normalized ratio
APTT: Activated partial thromboplastin time
TT: Thrombin time
FBG: Fibrinogen
DD: D-dimer
FDP: Fibrin degradation product
WBC: White blood cell
L1: Lymphocyte count
M1: Monocyte count
N1: Neutrophil count
E1: Eosinophil count
B1: Basophil count
RBC: Red blood cell
HGB: Hemoglobin
HCT: Hematocrit
RDW: Red blood cell distribution width
MCH: Mean corpuscular hemoglobin
MCV: Mean corpuscular volume
MCHC: Mean corpuscular hemoglobin concentration
PLT: Platelet count
MPV: Mean platelet volume
PCT: Plateletcrit
PDW: Platelet distribution width
DB: Direct bilirubin
IB: Indirect bilirubin
TP: Total protein
ALB: Albumin
ALT: Alanine aminotransferase
AST: Aspartate aminotransferase
ALP: Alkaline phosphatase
TBA: Total bile acid
GGT: γ-glutamyl transpeptidase
CHE: Cholinesterase
K: Potassium
Na: Sodium
Cl: Chloride
Ca: Calcium
CO2-CP: Carbon dioxide combining power
UREA: Blood urea nitrogen
CREA: Creatinine
CK-MB: Serum myocardial enzyme
cTnT: T cardiac troponin T
Myo: Myoglobin
NT-proBNP: N-terminal probrain natriuretic peptide
AUC: Area under the curve
ROC: Receiver operator characteristic curve
DCA: Decision curve analysis
